# MUM effect in medical education: taking into account the recipient and training setting

**DOI:** 10.1111/medu.13779

**Published:** 2018-12-12

**Authors:** Wendy E de Leng, Karen M Stegers‐Jager, Marise Ph Born, Axel P N Themmen

**Affiliations:** ^1^ Institute of Medical Education Research Rotterdam (iMERR) Erasmus MC Rotterdam the Netherlands; ^2^ Institute of Psychology Erasmus University Rotterdam Rotterdam the Netherlands

## Abstract

de Leng et al. argue that feedback communicators can be made more comfortable delivering unpleasant messages if clinical training settings promote trainees’ learning‐goal orientation.

In the process of becoming a medical doctor, receiving feedback from more experienced clinicians is essential. This feedback involves emphasising good performance, but also calling attention to poor performance. Theoretical and practical models have been described for effectively providing feedback in medical education.^1^ Despite the recognition of the relevance of feedback in clinical training, feedback providers struggle with delivering undesirable messages. Referring to this struggle, Scarff et al. turn to the literature on the MUM effect (keeping Mum about Unpleasant Messages) in this issue of *Medical Education*.^2^ The MUM effect indicates that individuals have more difficulty in delivering negative than positive messages, which often leads to avoiding or sugar‐coating the negative information. Our own research and that of others suggested that people agree more on what is considered undesirable, as opposed to desirable, because undesirable actions always lead to negative outcomes.^3^, ^4^ If it is so obvious to people when a behaviour or response is inappropriate, then why is it so difficult to express this seemingly apparent message? Scarff et al. attempt to answer this question by conducting a narrative review of the MUM literature.^2^ Three potential causes of the MUM effect are mentioned, namely avoidance of feelings of guilt and distress, avoidance of bad feelings in the recipient of the negative message and conformation to societal rules (e.g. politeness). All three causes refer to the communicator providing the feedback. By contrast with the feedback provider, we would like to focus this commentary on attributes of the feedback receiver by turning to the literature on goal orientation and achievement motivation. Subsequently, we discuss how social cues in the clinical training setting may affect receivers’ goal orientation and achievement motivation and thereby may help to ameliorate the MUM effect.

By contrast with the feedback provider, we would like to focus this commentary on attributes of the feedback receiver

What drives a person to learn? As a response to this question, Dweck (1986)^5^ made a distinction between learning and performance goals. Learning goals are aimed at mastering a new skill or ability, whereas performance goals are aimed at obtaining a favourable judgement of a skill or ability. Individuals who are fixated on getting a good judgement may be more reluctant to seek feedback until they know for sure that the required skill is achieved. By contrast, individuals who have a learning goal orientation are less apprehensive about showing that they have not yet mastered the required skill. The two types of goal orientations have been linked to beliefs about intelligence.^5^ Performance goals are often adopted by individuals who believe intelligence is a static entity that is hard to change. These people adhere to an ‘entity belief’. By contrast, learning goals are adopted by individuals who view intelligence as a malleable trait that can be developed (an incremental belief). It is not difficult to imagine that for a learner who is preoccupied by obtaining a good judgement about his or her ability level and who is convinced that it is hard or even impossible to change this ability level, negative feedback can be extremely disruptive. For these individuals, the obvious solution is to avoid seeking feedback, especially if the feedback is anticipated to be negative.^6^

… individuals who have a learning goal orientation are less apprehensive about showing that they have not yet mastered the required skill

The drive to learn can also be described by approach and avoidance achievement motivation.^7^ Individuals with a strong approach achievement motivation wish to be successful and to obtain a positive judgement. By contrast, individuals high on avoidance achievement motivation wish to avoid failure and a negative judgement. Although both types of performance goals impair feedback‐seeking behaviours, the impairment is greater for avoidance achievement motivation.^6^ Based on these theories, we argue that the MUM effect will be less strong if recipients of unpleasant messages predominantly hold a learning goal orientation, an incremental belief in the development of skills and abilities or an approach achievement motivation. Although these attributes are stable to some extent, they can be modified by social cues in a particular setting,^5^, ^6^, ^7^ for example an emphasis on competition with others and a tolerance of making mistakes. Awareness of these cues might help learners to feel more comfortable in receiving negative feedback and may indirectly lead communicators to feel more comfortable in providing negative feedback.

… we argue that the MUM effect will be less strong if recipients of unpleasant messages predominantly hold a learning goal orientation

Scarff et al. conclude their review with the proposition of several actions that may reduce the MUM effect in clinical training.^2^ Examples of these propositions are appointing a supervisor as an expert feedback provider who is trained to deliver negative messages, using multiple feedback providers to deliver a negative message, and reframing a negative message as useful information for development.^2^ In addition to these actions, we argue that attention should be directed to the social cues in the clinical training setting. Based on the literature on goal orientation and feedback‐seeking behaviours, we speculate that the promotion of a learning goal orientation in an organisation could ease the provision of negative feedback. Learning goals are encouraged by self‐referenced evaluation of performance and a tolerance of making mistakes, whereas performance goals are reinforced by other‐referenced evaluation of performance and an *in*tolerance of making mistakes.^6^ Supervisors with learning goals who focus on individual improvement instead of competition and who express that failure is part of the learning process provide students with sufficient resources to cope with setbacks.^8^ Unfortunately, cues that promote a learning goal orientation (i.e. self‐referenced evaluation and tolerance of mistakes) seem to be in conflict with the social cues in a clinical training setting. Firstly, in medical education, intense competition is the order of the day,^9^ fostering normative comparison with other students. Severe competition induces a strong tendency to obtain a relatively better evaluation of performance and to avoid a relatively worse evaluation of performance, impairing feedback‐seeking behaviours. Secondly, students in medical and clinical training settings experience several barriers to admitting medical errors or expressing uncertainties (e.g. the assumption that errors are a sign of incompetence).^10^ This intolerance of errors and insecurities generates a tendency to avoid failure and further reduces feedback‐seeking behaviours. Finally, we believe that the influence of social cues on the MUM effect should be viewed in interaction with cultural variables in the clinical training setting, such as power distance and individualism or collectivism.^11^

… the promotion of a learning goal orientation in an organisation could ease the provision of negative feedback

To summarise, in addition to the actions to ameliorate the MUM effect described by Scarff et al.,^2^ we highlight the significance of the characteristics of the recipient and of the social cues in the clinical training setting. We propose that a setting that de‐emphasises competition with others and that accepts making mistakes as part of the learning process may stimulate a learning goal orientation, which may subsequently reduce unpleasant feelings towards negative messages for both the recipient and communicator. Finally, we believe that awareness of these social cues is not only an additional action to reduce the MUM effect, but also constitutes a necessary condition for the effectiveness of the actions mentioned by Scarff et al.^2^

Unfortunately, cues that promote a learning goal orientation … seem to be in conflict with the social cues in a clinical training setting
